# Hepatic Plasmacytoma With DEL13q14 Positive on Fluorescent In Situ Hybridization (FISH) on Tissue Biopsy

**DOI:** 10.7759/cureus.33197

**Published:** 2022-12-31

**Authors:** Karthik Kumar, Sashikant Singh, Jhasaketan Nayak, Gaurav Dhingra, Uttam K Nath

**Affiliations:** 1 Department of Medical Oncology/Hematology, All India Institute of Medical Sciences, Rishikesh, IND

**Keywords:** del13q14, fish, hepatic, plasmacytoma, myeloma

## Abstract

A 40-year-old male presented with abdominal distension and dyspnea. On evaluation found to have hepatic plasmacytoma without marrow clonal plasma cells. Fluorescent in situ hybridization (FISH) on tissue biopsy revealed myeloma-defining cytogenetics. After treating with novel agents, the patient had a complete response to therapy.

## Introduction

Extramedullary plasmacytoma (EMP) is a rare presenting feature of multiple myeloma. EMP occurs in 7-18% of newly diagnosed myeloma and 20% of relapsed myeloma [1]. It commonly presents in the head and neck region. The cytogenetic abnormalities in multiple myeloma affect the disease evolution from malignancy to clinical presentation, response to therapy, and prognosis [2]. Here, we report a rare presentation of multiple myeloma presented as hepatic mass.

## Case presentation

A 40-year-old male presented with 1.5 years of fatigue and dyspnea, and abdominal distension for six months associated with significant weight loss. Eight months ago, the patient had a blunt injury over the abdomen and was diagnosed as hepatic contusion, and underwent surgery for the same. No other systemic complaints. On examination the patient was pale. Abdominal examination revealed a 5x5 cm mass in right hypochondrium and epigastrium without localizing signs (Table 1).

**Table 1:** Baseline investigations of the patient. AST: aspartate transaminase; ALT: alanine transaminase; PT: prothrombin time; aPTT: activated partial thromboplastin time; LDH: lactate dehydrogenase

Variables	Values
Hemoglobin	8.2 g/dL
Total leukocyte count	5530/mm^3^
Platelets	1.95 lakh/mm^3^
Bilirubin	0.81 mg/dL
AST/ALT	23/26 U/L
Albumin	1.92 g/dL
Urea/serum creatinine	37/1.29 mg/dL
Na^+^/K^+^/Ca^2+^	132/3.6/7.8 mg/dL
PT/aPTT	12 s/29 s
Serum LDH	118 (<248 U/L)

**Figure 1:**
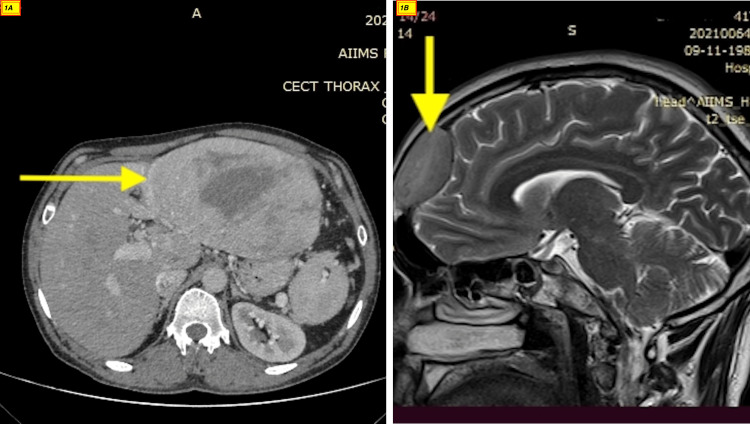
CT abdomen and MRI brain of the patient. The image shows a large lobulated altered signal intensity lesion measuring 122x76x148 mm in the epigastric region with non-separate visualization of pancreas and left liver lobe (A). An active lesion in the right frontal region of the brain with lytic changes in the frontal bone (B).

**Figure 2:**
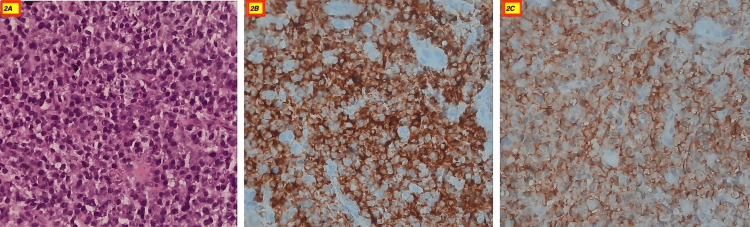
H&E stain (400x) clusters of plasma cells (A), plasma cells immunoreactive for CD38 (B), and plasma cells immunoreactive for CD138 (C). CD: cluster of differentiation

Ultrasonography showed a large heterogeneous hypoechoic lesion in the epigastric region abutting the liver with few periportal lymph nodes and a cystic lesion with septation at the splenic hilar region. Computed tomography showed a large lobulated altered signal intensity solid-cystic lesion measuring 122x76x148 mm in the epigastric region with non-separate visualization of the pancreas and left liver lobe and the lesion abutting the urinary bladder (Figure 1, panel A). The picture was more in favor of hepatocellular carcinoma. Serum alpha-fetoprotein (AFP) was 2 ng/mL (range: 0-8 ng/mL). The patient underwent a biopsy, which showed sheets of mature plasma cells with abundant dense cytoplasm, perinuclear hof, round eccentric nuclei, clock face chromatin, and indiscernible nucleoli (Figure 2, panel A) that are immunoreactive for cluster of differentiation (CD)38 (Figure 2, panel B), CD138 (Figure 2, panel C) and MUM1, and negative for pan-cytokeratin, carcinoembryonic antigen (CEA), CD34, CD20, desmin, and synaptophysin. With this clinical picture, plasma cell dyscrasia was considered. The workup for multiple myeloma was performed which included the bone marrow examination, fluorodeoxyglucose positron emission tomography (FDG PET-CT), and cytogenetic study.

**Figure 3:**
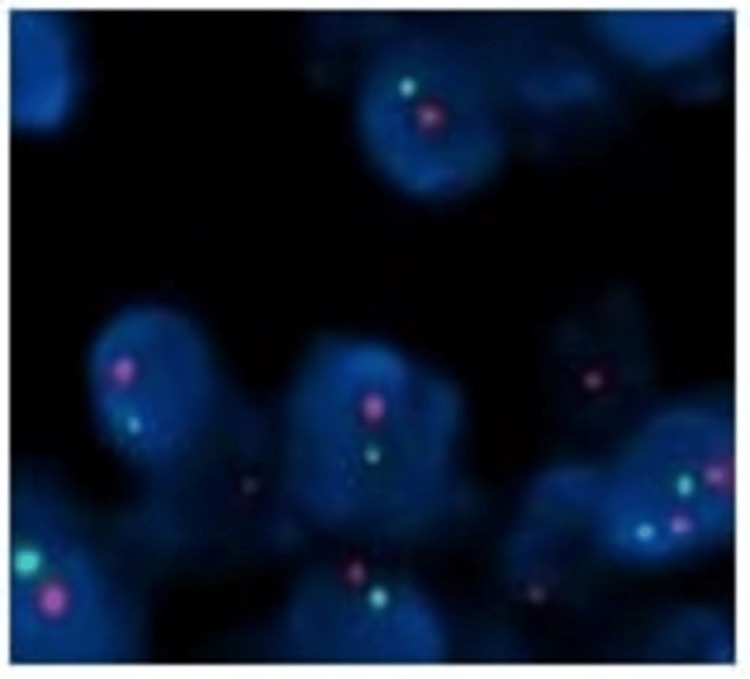
FISH on hepatic biopsy specimen showing DEL13q14.2/DEL13q34 using ZytoLight SPEC 13q14.2 spectrum orange/SPEC 13q34 spectrum green probe. FISH: fluorescent in situ hybridization

The bone marrow aspiration and biopsy showed normal cellular marrow with no abnormal plasma cells. The myeloma biochemical report revealed an M spike of 9.6 g/dL, IgG of 1234 mg/dL, kappa/lambda ratio of 42 (468/11), beta-2 microglobulin of 11.2 mg/L, and immunofixation showed IgG kappa band. With this, the diagnosis of hepatic plasmacytoma was confirmed. The FDG PET-CT was done, which showed multiple lesions in the abdomen involving the left lobe of the liver and other lesions in the periportal region abutting the duodenum and left hemidiaphragm abutting the spleen, and an active lesion in the right frontal region of the brain with lytic changes in the frontal bone. The same finding was confirmed with a contrast MRI brain and it measured 4.6x2.5x3.7 cm (Figure 1, panel B). The prognosis of myeloma depends on cytogenetics, and since the bone marrow didn’t show abnormal plasma cells, the interphase Fluorescent in situ hybridization (FISH) performed on the liver biopsy revealed DEL13q14 in 84% of the cells (Figure 3) [2].

He was started on triplet therapy with proteasome inhibitors (PI) and immunomodulators (IMiDs) (RVd - lenalidomide, bortezomib, and dexamethasone), and post 12 cycles of therapy, he attained a stringent complete response (no M spike, normalization of serum free light chain ratio, negative monoclonal immunofixation, and disappearance of plasmacytomas).

## Discussion

Multiple myeloma is defined by the International Myeloma Working Group (IMWG) as clonal bone marrow plasma cells ≥10% or biopsy-proven plasmacytoma plus one or more of the following myeloma-defining events: clonal plasma cells ≥60%, involved/uninvolved serum free light chain (SFLC) ratio ≥100, >1 focal lesion on MRI studies >5 mm; and hypercalcemia, renal failure, anemia, bone lytic lesions (CRAB) events (hypercalcemia, renal failure, anemia, and lytic bone lesions) [3]. Isolated EMP is a rare presenting feature of multiple myeloma. Very few cases of hepatic plasmacytoma have been reported [4-7]. The cytogenetic abnormalities in multiple myeloma affect the disease evolution from malignancy to clinical presentation, response to therapy, and prognosis. Since myeloma cells have a less proliferative capacity, interphase FISH is commonly used to detect cytogenetic abnormalities in myeloma rather than conventional metaphase karyotyping [8]. The challenge that remains in the diagnosis of isolated EMP's are the identification of primary and secondary cytogenetic abnormality in the tissue as the number of plasma cells can be less in the tissue biopsy as compared to bone marrow clonal plasma cells [9]. A minimum of 50 plasma cells (ideally 100 plasma cells) are required to detect the primary and secondary genetic abnormality. Interphase FISH is usually performed by sorting purified plasma cells (by CD138 magnetic beads) or labeling cytoplasmic immunoglobulin light chains to increase the number. As per the European Myeloma Network (EMN), the cut-off positivity for FISH is 10% for fusion or break-apart probes and 20% for numerical abnormalities [9]. Extramedullary involvement is one of the poor prognoses in multiple myeloma with high mortality and high relapse risk due to the presence of high-risk cytogenetics. EMP with newly diagnosed myeloma, EMP at relapse, CNS involvement, no high dose melphalan therapy followed by autologous stem cell transplant (ASCT), and International Staging System (ISS) II and III are associated with poor prognosis with EMP [10]. Our patient had hepatic plasmacytoma with CNS involvement with DEL13q14 abnormality which is non-high-risk myelomas cytogenetics and had a complete response to IMiDs and PI-based therapy.

## Conclusions

Isolated multiple EMP without bone marrow (BM) involvement is a rare presenting feature with multiple myeloma. The prognosis in these cases depends not only on the tumor biology but also on the presence of high-risk cytogenetics and the response to novel agents.
